# Supplementary material for: Effectiveness of physical rehabilitation interventions in critically ill patients—A protocol for an overview of systematic reviews
Source: PLoS One. 2023 Apr 13;18(4):e0284417. doi: 10.1371/journal.pone.0284417 (PMC10101388; doi:10.1371/journal.pone.0284417)
Supplement: Attachment — Submitted filename: Response to Reviewers.docx [file pone.0284417.s002.docx]

**Response to Reviewers**

| **Comments** | **Responses** |
| --- | --- |
| *Introduction*  I would like more context around the various populations and interventions that could be included here as the eligibility seems wide so coverage should be equally wide | Some ideas were added in the introduction to respond to the reviewer's suggestion. Based on this, some references were added, and the wording was modified |
| *Methods*  Are references 24 and 25 the best reference to use doesn’t it refer to a standard systematic review of interventions not an overview review? Doesn’t reference 26 cover what you want to do – how does reference 24 and 25 supplement 26? | Reference 24 points to the methodology, guidelines, or recommendations that we will use to conduct our overview (Cochrane Hanbook). Reference 25 points out that our protocol is reported following the PRISMA-P statement. And reference 26 points out that when we report the findings of our overview, we will use the PRIOR statement. The three references are not interchangeable but complementary |
| *Methods*  Can you justify why you are excluding some SRs with meta-analysis and add a reference | A sentence was added to justify the exclusion of revisions and a reference to substantiate |
| *Methods*  For eligibility around the type of intervention can you explain how you will deal with complex interventions that have multiple components where one is those you name but others are included | The following idea was added to the methods section to clarify the reviewer's stateme:  “Physical rehabilitation interventions may be delivered as a single intervention or in conjunction with other interventions (multimodal rehabilitation). However, interventions other than physical rehabilitation interventions should be delivered in a similar way in both groups (experimental and control) to isolate the effect of the physical interventions on the patient.” |
| *Methods*  Re AMSTAR May be worth contacting Lu et al 2020 for provisional findings from their work or obtain a reference to support choices made | Of interest is the study being conducted by Lu et al. We will watch for their results to be considered in discussing our results.  For now, we will continue to use the AMSTAR 2 tool to assess the quality of the included reviews, as it has proven to be a valid and reliable tool. |
| *Methods*  Can you identify if GRADE is designed to deal with a collection of systematic reviews rather than collection of interventions? Does the reader need to understand via a supplementary file what you will assess and how you will allocate points for upgrading and downgrading. I ma not clear how you will assess study inconsistency of results, indirectness of evidence, imprecision and reporting bias e.g., ROBIS is better than AMSTAR at reporting bias | In the methods section we specify that to report the certainty of evidence using the GRADE approach we will choose the "best" SR for each intervention and outcome. For this we will extract the analysis performed by the authors of the SRs, and if not performed by us we will do so.  The approach remains the same as for an SR of interventions. |
| *Methods*  Not convinced at how the analysis will account for different interventions, conditions, reviews with adults vs children may be a supplementary file could help this | The results of our overview will be presented in a narrative form using tables and figures. This will be done for all SRs, as well as separating them by population, intervention, and outcomes. This was explained in more detail in the methods section. |
| *Discussion*  Discussion is limited and I think you can expand based on the fact you have a very large and open consideration to all interventions and many population groups | A paragraph was added to the discussion to cover what was mentioned by the reviewer.  In addition, a paragraph was added on the potential limitations of our overview |
| *Title*  There are some grammatical issues with the topic of the topic. After the phrase critically ill patients, put a column and not a full stop. Start the next part with an article 'a' to read, 'A Protocol for an overview....' | The title was edited for clarity and to consider the reviewer's suggestion |
| *Introduction*  The sentence on line 72 which extends to line 74 should be reformulated to follow grammatical rules | Additions were made to the sentence mentioned by the reviewer, as well as to other sections of the manuscript, to improve clarity and comply with grammatical rules |
| *General comment*  You intend to review studies which look at both children and adults. Their musculo-skeletal adaptation is not the same will that not play on the outcome of this study? Is it not better to use just studies that include children or those involving just adults | This is true, so in the methods section, we explain that the analyses will also be performed and reported separately according to the population (adult vs. pediatric) |
